# Multi-Domain Neumann Network with Sensitivity Maps for Parallel MRI Reconstruction

**DOI:** 10.3390/s22103943

**Published:** 2022-05-23

**Authors:** Jun-Hyeok Lee, Junghwa Kang, Se-Hong Oh, Dong Hye Ye

**Affiliations:** 1 Department of Biomedical Engineering, Hankuk University of Foreign Studies, Yongin 17035, Korea; jhl22@hufs.ac.kr (J.-H.L.); jhkang@hufs.ac.kr (J.K.); 2 Department of Electrical and Computer Engineering, Marquette University, Milwaukee, WI 53323, USA

**Keywords:** magnetic resonance imaging, deep learning, Neumann network

## Abstract

MRI is an imaging technology that non-invasively obtains high-quality medical images for diagnosis. However, MRI has the major disadvantage of long scan times which cause patient discomfort and image artifacts. As one of the methods for reducing the long scan time of MRI, the parallel MRI method for reconstructing a high-fidelity MR image from under-sampled multi-coil k-space data is widely used. In this study, we propose a method to reconstruct a high-fidelity MR image from under-sampled multi-coil k-space data using deep-learning. The proposed multi-domain Neumann network with sensitivity maps (MDNNSM) is based on the Neumann network and uses a forward model including coil sensitivity maps for parallel MRI reconstruction. The MDNNSM consists of three main structures: the CNN-based sensitivity reconstruction block estimates coil sensitivity maps from multi-coil under-sampled k-space data; the recursive MR image reconstruction block reconstructs the MR image; and the skip connection accumulates each output and produces the final result. Experiments using the fastMRI T1-weighted brain image dataset were conducted at acceleration factors of 2, 4, and 8. Qualitative and quantitative experimental results show that the proposed MDNNSM method reconstructs MR images more accurately than other methods, including the generalized autocalibrating partially parallel acquisitions (GRAPPA) method and the original Neumann network.

## 1. Introduction

Magnetic resonance imaging (MRI) is one of the most-used medical imaging technologies. It is non-invasive and there is no radiation exposure, unlike X-ray and computed tomography (CT), so it is harmless to the human body. MRI follows the principle of nuclear magnetic resonance (NMR) to image the inside of the human body. It needs strong magnetic fields and electromagnetic waves to resonate hydrogen molecules in the human body, which either excites or relaxes them. Since the density of hydrogen molecules is different for each tissue, the intensity of the emitted signal varies with the scan parameters, such as the repetition time and the echo time. Therefore, by setting the scan parameters for a precise diagnosis, the suitable contrasts of the MR image can be acquired. The MR signal is transformed from the frequency domain to the spatial domain. Specifically, the MR signal collected by the radio frequency (RF) antenna is a complex value and is sampled in k-space. The k-space contains spatial frequency and phase information. After acquiring the k-space data, it is reconstructed into an MR image in the spatial domain using Fourier transform [1]. The conventionally used process for MRI scans has physical limits in terms of speed, because it has to be acquired sequentially in the k-space domain. Therefore, it requires a long scan time. In addition, the longitudinal relaxation time ($T_{1}$) increases as the external magnetic field strength increases. This results in an increase in scan time. Long scan times to acquire an MR image make patients uncomfortable. Moreover, artifacts are generated, depending on the patient’s movement or the uncontrollable flow of water molecules in the body (e.g., blood flow), during the MR scan. One of the methods used to reduce the long MR scan time is to obtain the k-space data by under-sampling. The under-sampling of an MR signal can reduce scan times because it acquires only a part of the k-space data. However, it also causes aliasing artifacts, due to the insufficient sampling rate.

Various methods have been proposed and developed in past years to reconstruct an artifact-free MR image from under-sampled data. One of those methods utilizes parallel imaging (PI) [2] to increase efficiency and accuracy in reconstruction. PI is a method that reconstructs under-sampled MR signals with coil sensitivities from multi-receiver RF coils to generate an artifact-free MR image. Multi-receiver RF coils have different sensitivity profiles, depending on their spatial location. Measuring the MR signal with multi-receiver RF coils is equivalent to performing additional sensitivity encoding. Multi-coil MR data with different spatial sensitivity profiles help the reconstruction process of mapping under-sampled k-space data to fully sampled MR images. The most important thing in PI is to effectively remove aliasing artifacts caused by violating the Nyquist theorem. In order to reconstruct an artifact-free MR image from multi-coil under-sampled k-space data, multiple methods have been proposed [3,4,5,6,7]. One method is to reconstruct an MR image using coil sensitivity maps in the image domain [3]. At this time, coil sensitivity maps are obtained in advance or are calculated from the acquired k-space data. Another method is to reconstruct an MR image by interpolating multi-coil data in the k-space domain and then combine the multi-coil data [5]. These PI methods are popular and widely used. However, it is challenging for conventional reconstruction methods to remove aliasing artifacts, particularly those with high acceleration factors such as 4 or 8.

In recent years, deep learning has been proposed in several studies for image restoration methods, such as for denoising [8,9,10], super-resolution [11,12,13,14], and inpainting [15,16,17], and it has been shown to work effectively. After that, model-based deep learning showed excellent performance by mathematically taking into account the image restoration operator as a forward model and solving the inverse problem to estimate the clean image for deep learning [18,19,20]. In particular, the Neumann network outperformed standard unrolled network architectures, such as model-based reconstruction with deep learned priors (MODL), by directly incorporating the forward model into the network optimization [19]. Moreover, deep learning-based methods show great promise in parallel MRI reconstruction when a high acceleration factor is used [21,22,23,24,25,26,27,28,29]. In addition, model-based deep learning, which sets parallel MRI reconstruction as an inverse problem and implements a forward model using prior knowledge, including coil sensitivity maps, shows excellent performance [30,31,32,33]. Coil sensitivity maps used in model-based deep learning for parallel MRI reconstruction are obtained in advance or calculated from auto-calibration signal (ACS) lines of the MR data, using an estimation method such as the ESPIRiT method [7]. Unfortunately, additional MR scanning to obtain coil sensitivity maps has the disadvantage of increasing the scan time, and estimation methods such as ESPIRiT have the disadvantage of low-accuracy estimations of sensitivity maps when the acceleration factor is high or when the ACS lines are few. Therefore, not only the MR image, but also the coil sensitivity maps, are necessary to estimate with deep learning [31,32].

In this study, we propose a multi-domain Neumann network with sensitivity maps (MDNNSM) that takes account into both image-domain and k-space-domain denoising, with the Neumann network’s regularization block combined with coil sensitivity maps. The MDNNSM consists of a data consistency block, a regularization block, and skip connections of the standard Neumann network, and an added sensitivity map reconstruction block. The new regularization block consists of two convolutional neural networks (CNNs) to reconstruct an MR image in the both image and frequency domains. The added sensitivity map reconstruction block maps multi-coil under-sampled k-space data to coil sensitivity maps for use in the forward model of parallel MRI reconstruction using CNNs. By integrating k-space data regularization and coil sensitivity map estimation into the Neumann Network, we achieved significant improvement in reconstruction quality when compared with the standard Neumann network [19].

## 2. Related Work

### 2.1. Parallel MRI Reconstruction Formulation

When acquiring MR signals, multi-receiver RF coils measure the signals in the frequency domain called the k-space. The MR image can be obtained by an inverse Fourier transform (IFT) of the acquired k-space data. Considering *y* is the measured under-sampled k-space data and *x* is the MR image that should be reconstructed, the process of scanning an MR image can be formulated as follows:

(1) y=Fx+ϵ,

where *F* is the Fourier transform (FT) and ϵ is the measurement noise. In parallel MRI, the MR scanner has multiple receiver RF coils, and each coil has a different sensitivity map, depending on its location. Each coil acquires signals in which the MR signal of *x* is affected by the coil’s sensitivity map as k-space data. Therefore, the MR signal acquisition in parallel MRI can be expressed as follows:

(2) A=M∘F∘S,

(3) y=Ax+ϵ

where *A* is a forward operator consisting of *F*, coil sensitivity maps *S*, and under-sampling mask operator *M*. With Equation (3) as an inverse problem, *x* can be obtained from the measured *y*. Unfortunately, since parallel MRI reconstruction is an ill-posed problem, there is no closed-form solution. So, the optimal solution is found using the optimization method that minimizes the least-squares problem as follows:

(4) $$x=\underset{x}{\mathrm{argmin}}\frac{1}{2}{∥Ax-y∥}_{2}^{2}+\lambda R\left(x\right)$$

where λ is a learning rate and R(·) is a regularization term. The regularizer *R* limits the degree of freedom of the solution by using prior knowledge about the MR image to be reconstructed. Classically, in the field of image reconstruction, total variation [34,35], $L_{1}$-norm wavelet transform [36], etc., are used. In MRI compression sensing (CS) [37], the $L_{1}$-norm wavelet transform is used as a regularization function to exploit image sparsity in the wavelet domain. Equation (4) can be solved by a gradient descent algorithm or a conjugate gradient algorithm. If it is solved using the gradient descent method, *x* is updated as follows in the *t*-th step.

(5) $$x^{t}=x^{t-1}-\lambda \left(A^{-1}\left(Ax^{t-1}-y\right)+R\left(x^{t-1}\right)\right)$$

Equation (5) calculates the approximate solution iteratively.

### 2.2. Deep Learning for Parallel MRI Reconstruction

In the past few years, many parallel MRI reconstruction methods using deep learning have been proposed [21,22,23,24,25,26,27,28,29]. Model-based deep learning architectures have recently gained popularity and achieved state-of-the-art performance [30,31,32,33]. This model-based method uses a regularizer *R* as a deep neural network in Equation (5) and reconstructs an MR image using an unrolled optimization.

(6) R(x)=CNN(x)

CNN-based regularization with a deep structure and nonlinear functions is also called data-driven regularization, and can approximate better than classical regularization. In addition, since CNNs learn the prior from the training data, the more data, the more the prior is helpful for the image reconstruction. For the model-based method, coil sensitivity maps *S* are used, and thus, need to be obtained or estimated. When coil sensitivity maps are obtained through additional MR scans, the scan time increases and the advantage of parallel imaging decreases. Another method is to estimate coil sensitivity maps from the acquired multi-coil k-space data. However, in conventional methods, such as ESPIRiT, as the acceleration factor increases, estimating accurate sensitivity maps becomes less likely. Recently, a method to reconstruct not only MR images, but also sensitivity maps by using deep learning has shown great promise [31,32].

### 2.3. Neumann Network

A Neumann network [19] is a deep neural network proposed to solve the inverse problem in the image processing field. Neumann series expansion is introduced to the inverse problem, and the solution is estimated by expanding as follows. The normal form of Equation (3) with the regularizer *R* is:

(7) $$x={\left(A^{-1}A+R\right)}^{-1}Ay$$

Using Neumann series expansion, Equation (7) can be expanded as:

(8) $$x=\sum_{j=0}^{\infty }{\left(I-\lambda A^{-1}A-\lambda R\right)}^{j}\left(\lambda A^{-1}y\right),$$

where λ represents trainable parameters. By truncating the series at *N*, we obtain:

(9) $$x=\sum_{j=0}^{N}{\left(I-\lambda A^{-1}A-\lambda R\right)}^{j}\left(\lambda A^{-1}y\right),$$

where the regularizer *R* is a trainable neural network. Equation (9) can be written in recursive form:

(10) $$x^{0}=\lambda A^{-1}y,$$

(11) $$x^{j}=x^{j-1}-\lambda A^{-1}Ax^{j-1}-R\left(x^{j-1}\right).$$

Then:

(12) $$\hat{x}=\sum_{j=0}^{N}x^{j}.$$

## 3. Multi-Domain Neumann Network with Sensitivity Maps

**Figure 1:**
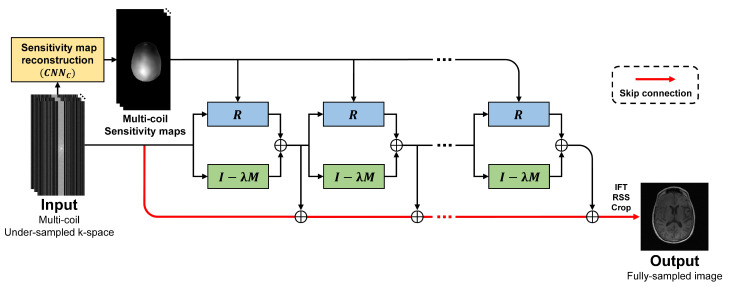
The overall architecture of the multi-domain Neumann network with sensitivity maps. The CNN-based sensitivity map reconstruction block reconstructs coil sensitivity maps from multi-coil under-sampled k-space. The block marked with (I−λM) is a data consistency block. The regularization block *R* reconstructs an MR image. The outputs of each iteration are accumulated in skip connections to become the final output.

Unlike the standard Neumann network, we propose a multi-domain Neumann network with sensitivity maps (MDNNSM), which incorporates coil sensitivity maps corresponding to multi-coil k-space data into a forward model for parallel MRI reconstruction. In this study, coil sensitivity maps are estimated and used with a deep learning-based method, instead of a conventional method such as ESPIRiT. Figure 1 illustrates the overall architecture of the MDNNSM. The network consists of a sensitivity map estimation block, a data consistency block, a regularization block, and skip connections. The sensitivity map estimation block estimates coil sensitivity maps from multi-coil k-space data using a CNN. the Data consistency and regularization blocks reconstruct an MR image using a forward model and a CNN. The skip connections accumulate outputs of each iterative and output them as the final output of the network.

### 3.1. Sensitivity Maps Estimation

Since we use the forward model of parallel MRI reconstruction in Equation (3) for the Neumann network, we estimate coil sensitivity maps from multi-coil k-space data, which is an input to the model. We use a CNN to reconstruct coil sensitivity maps, which can be rewritten as:

(13) $$S=CNN_{C}\left(F^{-1}\circ M_{ACS}\left(y\right)\right).$$

where $M_{ACS}$ is the ACS lines mask operator and $CNN_{C}$ is the CNN used to estimate the coil sensitivity maps. Except for low-frequency lines acquired by the ACS of multi-coil k-space data, zeros are filled in the other areas, transformed into an image domain by IFT, and then used as an input for $CNN_{C}$ [31]. Coil sensitivity maps estimated in this way are used for the forward model operation.

### 3.2. MR Image Reconstruction

**Figure 2:**
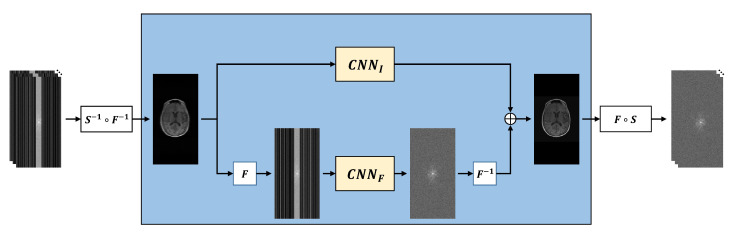
CNN-based regularization block. An MR image is reconstructed in parallel in the image domain and the k-space domain with two U-Nets and then added.

Based on Equation (11), an MR image is reconstructed using a data consistency block with the forward model and a CNN-based regularization block. Figure 2 illustrates the detail of the CNN-based regularization block. The CNN-based regularization block reconstructs an MR image in parallel with the regularization operating in both the image domain and the frequency domain. Using this, the CNN-based regularization *R* of Equation (11) is formulated as follows.

(14) $$R\left(x\right)=CNN_{I}\left(x\right)+F^{-1}CNN_{F}\left(Fx\right),$$

where $CNN_{I}$ and $CNN_{F}$ represent the CNN-based regularization that reconstructs an MR image in the image domain and the frequency domain, respectively. To reconstruct the MR image in the frequency domain, FT is applied to the MR image and used as the input of $CNN_{F}$; then, the IFT is applied to the output.

### 3.3. K-Space Domain Accumulation

The standard Neumann Network derives the final output by adding both the initial value and iteration outputs in the image domain [38]. Because the proposed MDNNSM handles data in the k-space domain (Equations (10)–(12)), the equation is updated by applying F∘S to both sides so that an MR image can be mapped to multi-coil k-space data.

(15) $$k^{0}=\lambda My,$$

(16) $$k^{j}=k^{j-1}-\lambda Mk^{j-1}-F\circ S\circ R\left(S^{-1}\circ F^{-1}k^{j-1}\right)\;\forall j=1,...,N,$$

(17) $$\hat{k}=\sum_{j=0}^{N}k^{j},$$

Finally, since the final output is the multi-coil k-space data, it is transformed into the image domain with IFT, and combined into an MR image with root sum squares:

(18) $$\hat{x}=\sqrt{\sum_{i=1}^{N}{∥F^{-1}\hat{k_{i}}∥}^{2}},$$

where $\hat{k_{i}}$ is the *i*-th coil k-space data of the final output.

## 4. Experiments

We compare our MDNNSM with the zero-filled method, the GRAPPA algorithm [5], U-Net [39], and Neumann networks [19]. Zero-filled MR images are used as the input of U-Net. Neumann networks reconstruct an MR image from multi-coil k-space data without considering the sensitivity map using CNN-based regularization in the image domain only. The reference image and the reconstruction images are normalized from 0 to 4095, and the difference map, depicting the difference between the reference image and the reconstruction image, is visualized. We evaluate MR images reconstructed by each method quantitatively, based on the normalized mean squared error (NMSE) and structural similarity (SSIM).

### 4.1. Implementation Details

We split the complex-valued k-space data into two channels, a real channel and an imaginary channel, and then concatenate them in the channel dimension to treat them as a real value. For example, 16-coil complex-valued data is treated as 32-channel data. Additionally, since the target ground truth is an MR image with a real value only, we calculate the loss between the target and the magnitude of the final output with a complex value.

In the MDNNSM, we use $CNN_{C}$ for the sensitivity map reconstruction and $CNN_{I}$ and $CNN_{F}$ for the MR image reconstruction. These three CNNs ($CNN_{C}$, $CNN_{I}$ and $CNN_{F}$) are implemented using same U-Net architecture [39]. U-Net consists of 2D convolution layers, leaky rectified linear units with a coefficient of 0.2 for negative values, and instance normalization [40]. We set the number of iteration blocks to 6 in the MDNNSM. The λ is initialized to 1 for all blocks. We use the SSIM loss function [41] and the ADAM optimizer [42] to train our network, with a learning rate of 0.0001 and 50 epochs.

**Figure 3:**
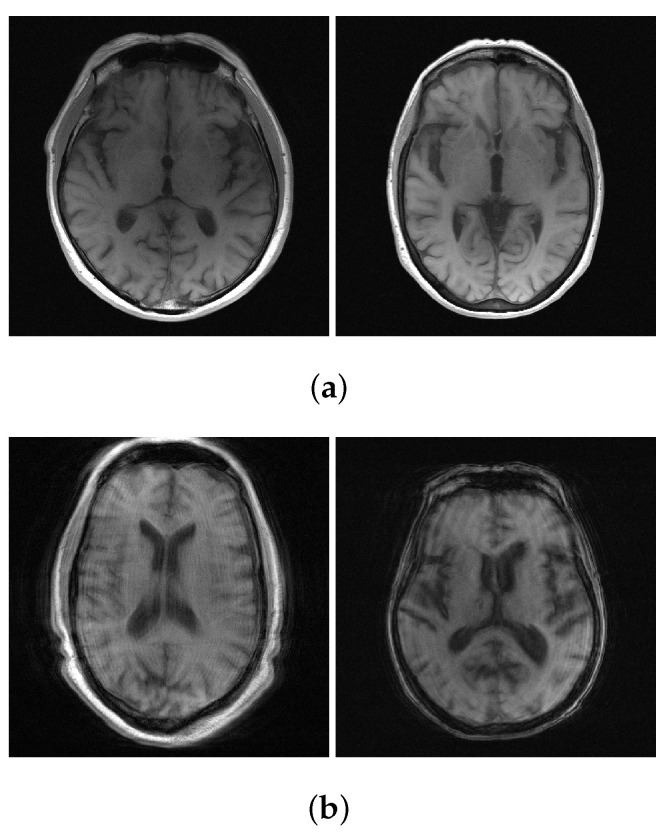
Examples of the NYU fastMRI brain dataset. (**a**) Artifact-free T1 weighted MR image; (**b**) Ringing artifact T1 weighted MR image.

**Figure 4:**
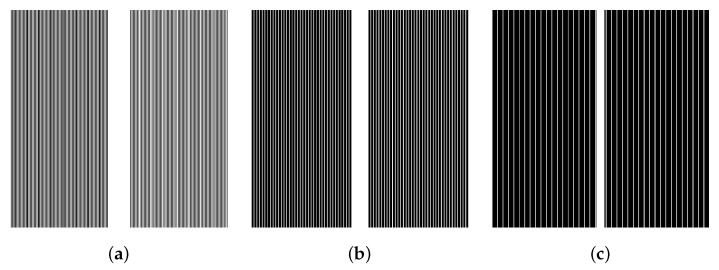
Examples of equi-spaced under-sampling masks with acceleration factors and ACS ratios of (**a**) (2, 0.1%), (**b**) (4, 0.08%), and (**c**) (8, 0.04%), respectively.

We used T1-weighted MR images of the NYU fastMRI brain dataset [43], which were obtained from four different MR scanners. The number of T1-weighted MR images is 498 scans (axial 7782 slices) for training and 169 scans (axial 2646 slices) for validation. The data used in our study subscribes to the following parameters: magnetic field strength = (1.5, 3) T, the number of multi-receiver coils = (2, 4, 6, 8, 12, 14, 16, 18, 20, 24), matrix size = ((640 × 260), (640 × 272), (640 × 290), (640 × 320), (640 × 332)), resolution = ((0.69 × 0.69), (0.69 × 0.72), (0.72 × 0.72), (0.75 × 0.75)) mm${}^{2}$, and slice thickness = (5, 7.5) mm. We used all 7782 slices for training; however, we conducted the validation without using the entire validation dataset, using only 1542 slices and excluding the data with ringing artifacts (as shown in Figure 3).To generate the multi-coil under-sampled k-space to be used for training and validation, multi-coil fully-sampled k-space data was multiplied pixel-wise by a sampling mask. As the sampling mask, equi-spaced sampling masks for regular under-sampling were used, as is shown in Figure 4. The acceleration factor and ACS ratio for sampling are as follows: (2, 10%), (4, 8%), and (8, 4%).

### 4.2. Results

**Figure 5:**
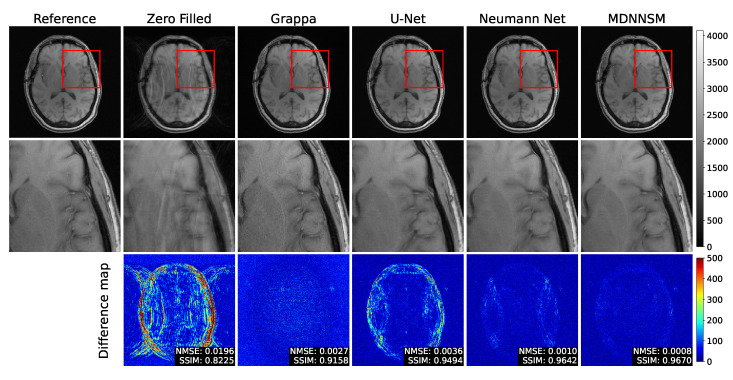
The top row shows fully sampled and reconstructed T1-weighted images using the zero-filled, Grappa, U-net, Neumann network, and MDNNSM methods, with an acceleration factor of 2 and an ACS rate of 10%. The middle row shows detailed images zooming the red box area of the top row. The bottom row shows the difference between the reference and reconstruction images. The MDNNSM showed the lowest NMSE and the highest SSIM.

In Figure 5, we present the fully sampled reference image and parallel MRI reconstruction images by various FFT-based and deep learning-based methods, with an acceleration factor of 2. The zero-filled image has severe aliasing artifacts due to the effect of under-sampling, making it difficult to observe the anatomy of the brain. When comparing reconstructed MR images of GRAPPA, U-Net, the Neumann network, and the MDNNSM, aliasing artifacts are removed, and the quality of reconstruction results is comparable to the fully sampled reference image.

**Figure 6:**
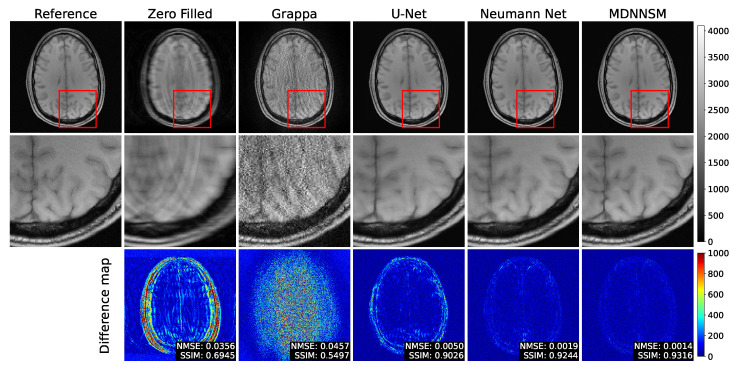
The top row shows fully sampled and reconstructed T1-weighted images using the zero-filled, Grappa, U-net, Neumann network, and MDNNSM methods, with an acceleration factor of 4 and an ACS rate 8%. The middle row shows detailed images zooming the red box area of the top row. The bottom row shows the difference between the reference and reconstruction images. The MDNNSM showed the lowest NMSE and the highest SSIM.

Figure 6 shows the fully-sampled reference image and the T1-weighted MR images reconstructed at an acceleration factor of 4 by each method. In the zero-filled image, there are more severe aliasing artifacts than in the result of Figure 5, due to the influence of the higher acceleration factor. For the same reason, the GRAPPA method also reconstructs the MR image with speckle noise. The U-Net method removes artifacts and noise well, but the image is blurred and details are lost. The Neumann network and the MDNNSM, which are model-based methods, have less blurring compared to the previous methods and reconstruct details well. In the difference map, the image reconstruction by the model-based method has a lower value, showing that it is reconstructed more similarly to the reference image. This indicates that incorporating a forward model into deep neural networks helps to improve fast MRI reconstruction.

**Figure 7:**
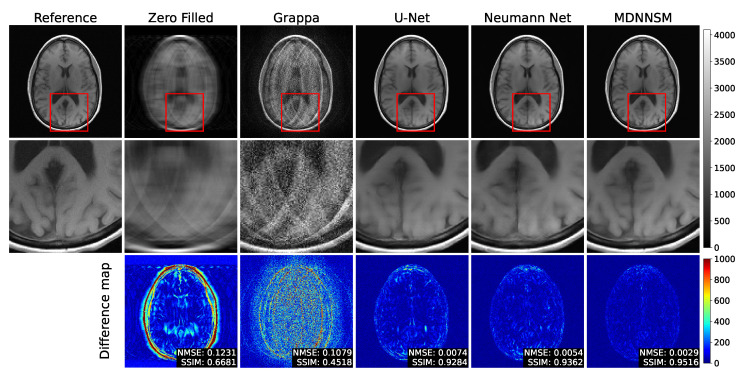
The top row shows fully sampled and reconstructed T1-weighted images using the zero-filled, Grappa, U-net, Neumann network, and MDNNSM methods, with an acceleration factor of 8 and an ACS rate 4%. The middle row shows detailed images zooming the red box area of the top row. The bottom row shows the difference between the reference and reconstruction images. The MDNNSM showed the lowest NMSE and the highest SSIM.

Figure 7 shows the reconstructed MR images of each method at an acceleration factor of 8. The analysis results of Figure 7 are similar to those of Figure 6. However, the quality of the reconstructed MR image is worse than the results of Figure 5 and Figure 6, due to the influence of a higher acceleration factor. In particular, MDNNSM significantly outperforms the standard Neumann network, as highlighted in the difference image. This shows the benefit of using CNN-based sensitivity map reconstruction.

We also report quantitative evaluation scores for parallel MRI reconstruction with acceleration factors of 2, 4, and 8 in Table 1. Our proposed MDNNSM produces significantly lower NMSE and higher SSIM than other reconstruction methods, including the original Neumann network.

### 4.3. The Amount of Data

**Figure 8:**
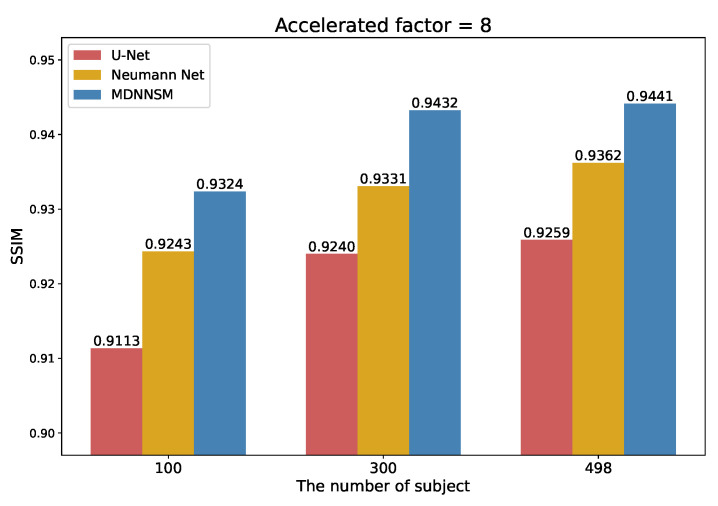
SSIM score according to the amount of training data at an acceleration factor of 8.

We compare the performance of the U-Net, Neumann network and MDNNSM methods according to the number of patient subjects used for training. Figure 8 shows the SSIM scores of the U-Net, Neumann network, and MDNNSM methods at an acceleration factor of 8 when the number of training subjects is 100, 300, and 498. Regardless of the number of data, the performance is good in the order of the MDNNSM, the Neumann network, and U-Net. When comparing the MDNNSM and U-Net, the increase in SSIM score is 0.0211, 0.0192, and 0.0182 for 100, 300, and 498 training subjects, respectively. This indicates that the MDNNSM, using the forward model, is robust even when trained with less data than U-Net.

### 4.4. Ablation Studies

To evaluate the efficacy of the network architecture, we evaluated the MDNNSM by implementing its architecture depending on the following three points:

1 Sensitivity maps estimation: A comparison of the performance according to the sensitivity maps estimated by ESPIRiT or $CNN_{C}$;
2 Accumulating domain: A comparison of the performance according to the domain where data accumulates in the skip connections, either in the image domain or the frequency domain;
3 Sharing network parameters: A comparison of the performance according to paramaters that were shared with U-Net for the CNN-based regularization block in each iteration;

#### 4.4.1. Sensitivity Maps Estimation

Table 2 shows the quantitative performance comparison of MR image reconstruction according to the sensitivity map estimation method. The MR image reconstruction performance of the MDNNSM is better when using sensitivity maps estimated by $CNN_{C}$ than when using sensitivity maps estimated by the ESPIRiT method. In all cases of acceleration factors 2, 4, and 8, when sensitivity maps estimated by $CNN_{C}$ were used, the NMSE was lower and the SSIM was higher.

#### 4.4.2. Accumulating Domain

Table 3 shows the performance of each case quantitatively when data were accumulated in the image domain and the k-space domain in the skip connections of the MDNNSM. When data was accumulated in the k-space domain at acceleration factors of 2 and 4, it showed higher NMSE and SSIM scores than the results using accumulated data in the image domain. When comparing the results from using a factor of 8, the results of the image domain scored lower errors in NMSE than the results of the k-space domain. However, for SSIM, the results of the k-space domain were higher than image domain.

#### 4.4.3. Sharing the Network Parameters

Table 4 shows a quantitative comparison of the performance according to shared parameters in the MDNNSM. Sharing the parameters means sharing a CNN-based regularization block in each iteration of the MDNNSM. If the parameters are shared in a regularization block for every iteration, the input is reconstructed by the CNN with the same weights. In all cases where acceleration factors of 2, 4, and 8, were used, the performance was good for the MDNNSM without parameter sharing. Furthermore, as the acceleration factor increased, the performance difference between the two cases increased as well.

## 5. Discussion

In this study, we introduced a method for parallel MRI reconstruction from under-sampled multi-coil k-space data. An MDNNSM, based on the Neumann network and with two added elements, was implemented.

First, a CNN was added for estimating coil sensitivity maps and used to perform the parallel MRI forward model operation. One method to obtain coil sensitivity maps without deep learning is to obtain the standard coil sensitivity map using full-scan data. The standard coil sensitivity maps obtained by this method have the advantage of high sensitivity profile accuracy, but because full-scan data need to be acquired, the advantage of accelerated MRI that measures MR signals by under-sampling disappears. Another method is to derive coil sensitivity maps from ACS lines, such as with the ESPIRiT method. In the self-calibrated coil sensitivity maps, the accuracy of the sensitivity profile tends to decrease as the number of ACS lines decreases. The accuracy of the low sensitivity profile may lower the quality of the reconstructed MR image. Therefore, we designed not only MR image reconstruction but also coil sensitivity map estimation with a CNN to increase the accuracy of the sensitivity profile. Consequently, we automated the process of reconstructing an MR image from multi-coil under-sampled k-space data, and, as shown in Table 2, the resulting reconstructed MR images were better than those reconstructed using ESPIRiT.

Second, in CNN-based regularization, multi-domain regularization was used for reconstruction in both the image and frequency domains. Using multi-domain regularization, aliasing artifacts were removed in the image domain and interpolation was performed by estimating missing data in the frequency domain. As a result of implementing three acceleration factors, such as 2, 4, and 8, the MDNNSM outperformed the reconstruction performance of the original Neumann network and other state-of-the-art methods. The aliasing artifacts were reduced and detailed structures were reconstructed.

The MDNNSM reconstructs an MR image by directly incorporating the forward model into the network optimization. Instead of reconstructing an MR image without an explicit formula, we took an MRI-specific approach, using the forward model of parallel MRI. With this approach, we were able to apply the prior knowledge of parallel MRI acquisitions to the deep neural network and to improve the quality of the reconstructed MR image. Moreover, by applying the forward model using estimated sensitivity maps, multi-coil data could be mapped into single data. Accordingly, not only the reconstruction accuracy was increased, but the size of the calculated data was also reduced, so the memory efficiency was also improved.

In the current study, only T1-weighted brain images from the fastMRI dataset were used. MR images can have various modalities, as well as T1 weights, depending on their purpose. Therefore, it is necessary to evaluate the MDNNSM using various data in future studies. The reconstruction performance of the MDNNSM will be evaluated for various MR images, such as T2-weighted images or fluid attenuated inversion recovery (FLAIR) images. In addition, since the accuracy of the estimated sensitivity maps has a great effect on the MR image reconstruction performance, it is necessary to study and apply a method for estimating the sensitivity maps with high accuracy.

## 6. Conclusions

We proposed an MDNNSM for parallel MRI reconstruction by incorporating multi-domain regularization and coil sensitivity map estimation into a Neumann network. Experimental results demonstrated that our MDNNSM shows superior reconstruction quality, compared with other FFT-based and deep learning-based parallel MRI reconstruction methods—including even the original Neumann network. This means that, when parallel MRI reconstruction is performed, using a forward model with sensitivity maps increases the accuracy of the reconstructed MR image.

## Figures and Tables

**Table 1:** Quantitative evaluation of reconstructed T1-weighted images with acceleration factors of 2, 4, and 8.

Model	2X Acceleration Factor		4X Acceleration Factor		8X Acceleration Factor	
	NMSE	SSIM	NMSE	SSIM	NMSE	SSIM
Zero-filled	0.0133	0.9084	0.0360	0.8079	0.0913	0.7003
GRAPPA	0.0049	0.9247	0.0584	0.6823	0.0939	0.5848
U-Net	0.0020	0.9696	0.0045	0.9501	0.0102	0.9259
Neumann network	0.0013	0.9737	0.0028	0.9579	0.0069	0.9362
MDNNSM	**0.0012**	**0.9747**	**0.0023**	**0.9612**	**0.0051**	**0.9441**

**Table 2:** Quantitative evaluation of reconstructed T1-weighted images depending on the sensitivity map estimation method.

Model	2X Acceleration Factor		4X Acceleration Factor		8X Acceleration Factor	
	NMSE	SSIM	NMSE	SSIM	NMSE	SSIM
MDNNSM with ESPRiT	0.0014	0.9711	0.0030	0.9514	0.0068	0.9268
MDNNSM with $CNN_{C}$	**0.0012**	**0.9747**	**0.0023**	**0.9612**	**0.0051**	**0.9441**

**Table 3:** Quantitative evaluation of reconstructed T1-weighted images depending on the accumulating domain.

Model	2X Acceleration Factor		4X Acceleration Factor		8X Acceleration Factor	
	NMSE	SSIM	NMSE	SSIM	NMSE	SSIM
MDNNSM Image sum	0.0014	0.9710	0.0024	0.9599	**0.0050**	0.9439
MDNNSM K-space sum	**0.0012**	**0.9747**	**0.0023**	**0.9612**	0.0051	**0.9441**

**Table 4:** Quantitative evaluation of reconstructed T1-weighted images depending on the shared parameters.

Model	2X Acceleration Factor		4X Acceleration Factor		8X Acceleration Factor	
	NMSE	SSIM	NMSE	SSIM	NMSE	SSIM
MDNNSM with parameter sharing	0.0013	0.9745	0.0025	0.9600	0.0060	0.9404
MDNNSM without parameter sharing	**0.0012**	**0.9747**	**0.0023**	**0.9612**	**0.0051**	**0.9441**

## Data Availability

Data used in the preparation of this article were obtained from the NYU fastMRI Initiative database (https://fastmri.med.nyu.edu/) (accessed on 13 April 2022) [43,44]. As such, NYU fastMRI investigators provided data but did not participate in the analysis or writing of this report. A listing of NYU fastMRI investigators, subject to updates, can be found at: https://fastmri.med.nyu.edu/ (accessed on 13 April 2022). The primary goal of fastMRI is to test whether machine learning can aid in the reconstruction of medical images.

## References

[1] Paschal C.B., Morris H.D. (2004). K-space in the clinic. J. Magn. Reson. Imaging.

[2] Heidemann R.M., Özsarlak Ö., Parizel P.M., Michiels J., Kiefer B., Jellus V., Müller M., Breuer F., Blaimer M., Griswold M.A. (2003). A brief review of parallel magnetic resonance imaging. Eur. Radiol..

[3] Pruessmann K.P., Weiger M., Scheidegger M.B., Boesiger P. (1999). SENSE: Sensitivity encoding for fast MRI. Magn. Reson. Med..

[4] Ying L., Sheng J. (2007). Joint image reconstruction and sensitivity estimation in SENSE (JSENSE). Magn. Reson. Med..

[5] Griswold M.A., Jakob P.M., Heidemann R.M., Nittka M., Jellus V., Wang J., Kiefer B., Haase A. (2002). Generalized autocalibrating partially parallel acquisitions (GRAPPA). Magn. Reson. Med..

[6] Lustig M., Pauly J.M. (2010). SPIRiT: Iterative self-consistent parallel imaging reconstruction from arbitrary k-space. Magn. Reson. Med..

[7] Uecker M., Lai P., Murphy M.J., Virtue P., Elad M., Pauly J.M., Vasanawala S.S., Lustig M. (2014). ESPIRiT—An eigenvalue approach to autocalibrating parallel MRI: Where SENSE meets GRAPPA. Magn. Reson. Med..

[8] Zhang K., Zuo W., Chen Y., Meng D., Zhang L. (2017). Beyond a gaussian denoiser: Residual learning of deep cnn for image denoising. IEEE Trans. Image Process..

[9] Zhang K., Zuo W., Gu S., Zhang L. Learning deep CNN denoiser prior for image restoration. Proceedings of the IEEE Conference on Computer Vision and Pattern Recognition.

[10] Tian C., Xu Y., Li Z., Zuo W., Fei L., Liu H. (2020). Attention-guided CNN for image denoising. Neural Netw..

[11] Dong C., Loy C.C., He K., Tang X. (2015). Image super-resolution using deep convolutional networks. IEEE Trans. Pattern Anal. Mach. Intell..

[12] Lai W.S., Huang J.B., Ahuja N., Yang M.H. Deep laplacian pyramid networks for fast and accurate super-resolution. Proceedings of the IEEE Conference on Computer Vision and Pattern Recognition.

[13] Yamanaka J., Kuwashima S., Kurita T. (2017). Fast and accurate image super resolution by deep CNN with skip connection and network in network. Proceedings of the International Conference on Neural Information Processing.

[14] Zhang Y., Li K., Li K., Wang L., Zhong B., Fu Y. Image super-resolution using very deep residual channel attention networks. Proceedings of the European Conference on Computer Vision (ECCV).

[15] Yan Z., Li X., Li M., Zuo W., Shan S. Shift-net: Image inpainting via deep feature rearrangement. Proceedings of the European Conference on Computer Vision (ECCV).

[16] Zeng Y., Fu J., Chao H., Guo B. Learning pyramid-context encoder network for high-quality image inpainting. Proceedings of the IEEE/CVF Conference on Computer Vision and Pattern Recognition.

[17] Pathak D., Krahenbuhl P., Donahue J., Darrell T., Efros A.A. Context encoders: Feature learning by inpainting. Proceedings of the IEEE Conference on Computer Vision and Pattern Recognition.

[18] Aggarwal H.K., Mani M.P., Jacob M. (2018). MoDL: Model-based deep learning architecture for inverse problems. IEEE Trans. Med. Imaging.

[19] Gilton D., Ongie G., Willett R. (2019). Neumann networks for linear inverse problems in imaging. IEEE Trans. Comput. Imaging.

[20] Diamond S., Sitzmann V., Heide F., Wetzstein G. (2017). Unrolled optimization with deep priors. arXiv.

[21] Schlemper J., Caballero J., Hajnal J.V., Price A.N., Rueckert D. (2017). A deep cascade of convolutional neural networks for dynamic MR image reconstruction. IEEE Trans. Med. Imaging.

[22] Eo T., Shin H., Jun Y., Kim T., Hwang D. (2020). Accelerating Cartesian MRI by domain-transform manifold learning in phase-encoding direction. Med. Image Anal..

[23] Wang S., Su Z., Ying L., Peng X., Zhu S., Liang F., Feng D., Liang D. (2016). Accelerating magnetic resonance imaging via deep learning. Proceedings of the 2016 IEEE 13th International Symposium on Biomedical Imaging (ISBI).

[24] Du T., Zhang H., Li Y., Pickup S., Rosen M., Zhou R., Song H.K., Fan Y. (2021). Adaptive convolutional neural networks for accelerating magnetic resonance imaging via k-space data interpolation. Med. Image Anal..

[25] Lee D., Yoo J., Tak S., Ye J.C. (2018). Deep residual learning for accelerated MRI using magnitude and phase networks. IEEE Trans. Biomed. Eng..

[26] Tavaf N., Torfi A., Ugurbil K., Van de Moortele P.F. (2021). GRAPPA-GANs for Parallel MRI Reconstruction. arXiv.

[27] Eo T., Jun Y., Kim T., Jang J., Lee H.J., Hwang D. (2018). KIKI-net: Cross-domain convolutional neural networks for reconstructing undersampled magnetic resonance images. Magn. Reson. Med..

[28] Han Y., Sunwoo L., Ye J.C. (2019). k-space deep learning for accelerated MRI. IEEE Trans. Med. Imaging.

[29] Sriram A., Zbontar J., Murrell T., Zitnick C.L., Defazio A., Sodickson D.K. GrappaNet: Combining parallel imaging with deep learning for multi-coil MRI reconstruction. Proceedings of the IEEE/CVF Conference on Computer Vision and Pattern Recognition.

[30] Hammernik K., Klatzer T., Kobler E., Recht M.P., Sodickson D.K., Pock T., Knoll F. (2018). Learning a variational network for reconstruction of accelerated MRI data. Magn. Reson. Med..

[31] Sriram A., Zbontar J., Murrell T., Defazio A., Zitnick C.L., Yakubova N., Knoll F., Johnson P. (2020). End-to-end variational networks for accelerated MRI reconstruction. Proceedings of the International Conference on Medical Image Computing and Computer-Assisted Intervention.

[32] Jun Y., Shin H., Eo T., Hwang D. Joint deep model-based MR image and coil sensitivity reconstruction network (joint-ICNet) for fast MRI. Proceedings of the IEEE/CVF Conference on Computer Vision and Pattern Recognition.

[33] Putzky P., Karkalousos D., Teuwen J., Miriakov N., Bakker B., Caan M., Welling M. (2019). i-RIM applied to the fastMRI challenge. arXiv.

[34] Block K.T., Uecker M., Frahm J. (2007). Undersampled radial MRI with multiple coils. Iterative image reconstruction using a total variation constraint. Magn. Reson. Med..

[35] Knoll F., Bredies K., Pock T., Stollberger R. (2011). Second order total generalized variation (TGV) for MRI. Magn. Reson. Med..

[36] Donoho D.L. (2006). Compressed sensing. IEEE Trans. Inf. Theory.

[37] Lustig M., Donoho D.L., Santos J.M., Pauly J.M. (2008). Compressed sensing MRI. IEEE Signal Process. Mag..

[38] He K., Zhang X., Ren S., Sun J. Deep residual learning for image recognition. Proceedings of the IEEE Conference on Computer Vision and Pattern Recognition.

[39] Ronneberger O., Fischer P., Brox T. (2015). U-net: Convolutional networks for biomedical image segmentation. Proceedings of the International Conference on Medical Image Computing and Computer-Assisted Intervention.

[40] Ulyanov D., Vedaldi A., Lempitsky V. (2016). Instance normalization: The missing ingredient for fast stylization. arXiv.

[41] Wang Z., Bovik A.C., Sheikh H.R., Simoncelli E.P. (2004). Image quality assessment: From error visibility to structural similarity. IEEE Trans. Image Process..

[42] Kingma D.P., Ba J. (2014). Adam: A method for stochastic optimization. arXiv.

[43] Zbontar J., Knoll F., Sriram A., Murrell T., Huang Z., Muckley M.J., Defazio A., Stern R., Johnson P., Bruno M. (2018). fastMRI: An open dataset and benchmarks for accelerated MRI. arXiv.

[44] Knoll F., Zbontar J., Sriram A., Muckley M.J., Bruno M., Defazio A., Parente M., Geras K.J., Katsnelson J., Chandarana H. (2020). fastMRI: A publicly available raw k-space and DICOM dataset of knee images for accelerated MR image reconstruction using machine learning. Radiol. Artif. Intell..

